# Uranium (U)-Tolerant Bacterial Diversity from U Ore Deposit of Domiasiat in North-East India and Its Prospective Utilisation in Bioremediation

**DOI:** 10.1264/jsme2.ME12074

**Published:** 2012-10-19

**Authors:** Rakshak Kumar, Macmillan Nongkhlaw, Celin Acharya, Santa Ram Joshi

**Affiliations:** 1 Microbiology Laboratory, Department of Biotechnology & Bioinformatics, North-Eastern Hill University, Umshing, Shillong, Meghalaya 793 022, India; 2 Molecular Biology Division, BioMedical Group, Bhabha Atomic Research Centre, Trombay, Mumbai, Maharashtra 400 085, India

**Keywords:** Domiasiat bacterial diversity, uranium tolerance and binding, phosphatase, P_IB_-type ATPase gene, bioremediation

## Abstract

Uranium (U)-tolerant aerobic chemo-heterotrophic bacteria were isolated from the sub-surface soils of U-rich deposits in Domiasiat, North East India. The bacterial community explored at molecular level by amplified ribosomal DNA restriction analysis (ARDRA) resulted in 51 distinct phylotypes. Bacterial community assemblages at the U mining site with the concentration of U ranging from 20 to 100 ppm, were found to be most diverse. Representative bacteria analysed by 16S rRNA gene sequencing were affiliated to *Firmicutes* (51%), *Gammaproteobacteria* (26%), *Actinobacteria* (11%), *Bacteroidetes* (10%) and *Betaproteobacteria* (2%). Representative strains removed more than 90% and 53% of U from 100 μM and 2 mM uranyl nitrate solutions, respectively, at pH 3.5 within 10 min of exposure and the activity was retained until 24 h. Overall, 76% of characterized isolates possessed phosphatase enzyme and 53% had P_IB_-type ATPase genes. This study generated baseline information on the diverse indigenous U-tolerant bacteria which could serve as an indicator to estimate the environmental impact expected to be caused by mining in the future. Also, these natural isolates efficient in uranium binding and harbouring phosphatase enzyme and metal-transporting genes could possibly play a vital role in the bioremediation of metal-/radionuclide-contaminated environments.

Uranium (U) is a ubiquitous element and traces of U occur almost everywhere, rarely occurring as rich deposits in the earth’s crust. In India, one such rich deposit includes the phanerozoic sandstone-type deposits of Kylleng-Pyndengsohiong (formerly known as Domiasiat and Wahkyn) in the West Khasi hills district of Meghalaya (35, 40). Domiasiat deposit is host by Kylleng and Rangam blocks with peneconcordant, tabular sandstone-type U mineralization and *in situ* resources of the order of 8,000 tonnes at grades averaging 0.1% U, making this U deposit so far the largest of its kind in India (39). Overburden (soil surface above the U rock) is from a few meters to over 25 m thick with an ore to overburden ratio of 1:6.7, and this deposit is amenable to open cast mining (45 m deep) (8). The indigenous microbial communities from such U ore deposits have been explored less than the relatively broader assessment of the microbial community structure in copper and gold mines or in metal-/radionuclide-contaminated environments (10, 36, 41).

Highly sensitive ecological parameters including microbial community composition, metal resistance and microbial diversity indices are increasingly being considered as effective tools to determine the impact of environmental contamination (9, 20). It is important to have information on the physiology of pure microbial organisms recovered or related to the natural populations (26), and hence, there is a need to isolate bacteria from U-contaminated sites and to study their interactions with U, which occur through various mechanisms, including biosorption at the cell surface, intracellular accumulation, precipitation, and redox transformations (oxidation/reduction) (30). The identification and documentation of a U-tolerant pure bacterial culture from an ore deposit site with the possibility of mining in the near future may serve as baseline information to gauge the subsequent impact of environment perturbation. Extensive investigation of the taxonomic position of microorganisms surviving in U ore deposits, such as in Domiasiat, and studying their interaction with U and other metals can provide vital background information on bioremediation approaches.

Numerous studies have investigated microbial communities in mine tailings, mine wastes, mining-impacted sites, and other U-contaminated subsurface environments utilising 16S rRNA gene retrieval and phylogenetic placement (1, 13, 36, 38). In contrast, the microbial community within subsurface U ore remains unexplored and uncharacterized despite their inevitable presence and role in mineral biogeochemistry (19). The present study aimed at the molecular characterization of bacterial diversity from one such subsurface pre-mined U ore deposits of Meghalaya and was basically designed with the view i) to obtain baseline information on cultivated pure indigenous U-tolerant bacterial communities which could serve as indicator to estimate the impact of mining to be undertaken in the future and (ii) to study their interaction with U to explore their potential as bioremediation agents against U-contaminated waste.

## Materials and Methods

### Sampling and enumeration of viable bacteria

Soil samples were collected from the area around the proposed mining site of U ore deposit in triplicate at a depth of 15–30 cm in sterile plastic bags and transported on ice to the laboratory. The global positioning system (GPS) coordinates of the sampling points are displayed in Table 1 where KMS1 stands for Kylleng Mining Site 1, PB1 for Photkylleng Block 1, PMS for Phudsyngkai Mining Site, KMS1A2TS for KMS1 A2 Tailing Site, KMS1DrP for KMS1 Drilling point, BIA for Block I Area, KMS1A2OT for KMS1 A2 Ore Tanks, PV for Pyndengsohiong Village, LV for Longorli village and WJ for Wahkhaji. The concentration of U was analysed using an inductively coupled plasma spectrometer (ICP-MS, Elan DRC; Perkin Elmer) and the trace metals were measured using an atomic absorption spectrophotometer (AAS, 3110; Perkin Elmer). Before analysis, acid digestion of the soil samples using nitric acid (HNO_3_) was performed in order to completely transfer the analytes into a solution so that they could undergo the determination step in liquid form. Sampling points were subsequently grouped into three categories based on the observed soil U concentrations (Table 1). Serial tenfold dilution of soil samples were made with 0.85% saline to plate in triplicate on R2A agar media (pH 7.0; Himedia, India) (22) and incubated at 30°C for 72 h. Viable aerobic chemo-heterotrophic bacteria obtained from the agar plates were counted as colony forming units (CFU) and expressed as CFU g^−1^ dry weight of soil.

### Screening and isolation of subsurface U-tolerant bacteria

Duplicate soil samples (10 g) were inoculated in 100 mL low phosphate medium (LPM; pH 7.5) (33) in Erlenmeyer flasks containing 1 mM U(VI) as UO_2_(NO_3_)_2_·6H_2_O and incubated at 30°C at 150 rpm for 48 h. Serial ten-fold dilutions of these enrichment cultures were inoculated onto LPM agar (pH 7.0) plates (22) supplemented with 1 mM U(VI) to isolate U-tolerant populations. Plates were incubated at 30°C for 72 h. A total of 130 U-tolerant bacterial colonies were randomly picked up from the plates for identification and further study for their relevance in bioremediation. The purity of the cultures was confirmed by the streak plate method using nutrient agar medium and they were preserved using 15% glycerol at −70°C. Gram staining and the cell shape were observed on microscopic studies using a Leica DM 1000 inverted light microscope (Leica Microsystems, USA).

### Nucleic acid extraction and PCR amplification

Genomic DNA from the selected 130 metal-tolerant bacteria was extracted using a HiPurA Bacterial and Yeast Genomic DNA Miniprep Purification Spin Kit (HiMedia, India). Then, 16S rRNA gene sequences were amplified by PCR using two general bacterial 16S rRNA gene primers. PCR mixtures (25 μL) contained approximately 30 ng template DNA, 2 μM forward primer 27F (5′-AGAGTTTGATCCTGGCTCAG-3′), 2 μM reverse primer 1492R (5′-TACGGYTACCTTGTTACGACTT- 3′), 1.5 mM of MgCl_2_ (Taq Buffer), deoxynucleoside triphosphates (250 μM each of dATP, dCTP, dGTP and dTTP) and 0.6 U Taq polymerase. DNA amplification was carried out using the GeneAMP PCR system 9700 (Applied Biosystems, CA, USA) and approximately l,500 bp were amplified. Templates replaced with sterile water were always used as negative controls.

### Fingerprinting of the 16S rRNA gene with amplified ribosomal DNA restriction analysis (ARDRA) and sequence analysis of 16S rRNA gene

The PCR-amplified 16S rRNA gene products (10 μL) were digested separately with two frequently cutting endonucleases 1U *Msp*I and 1U *Hae*III for 4 h at 37°C according to the manufacturer’s instructions (Fermentas, Canada) in a 20 μL reaction mixture to determine the characteristic ARDRA pattern of each isolate. The reaction was stopped by thermal inactivation at 65°C for 20 min. After digestion, the resulting products were separated by electrophoresis on a 3% agarose gel at 80 V and by ethidium bromide staining. Restriction patterns were analysed visually using a Transilluminator (UVITEC, UK) to group the isolates based on banding patterns. Two isolates were considered members of the same phylotype when their ARDRA patterns were identical for both restriction enzymes. The 16S rRNA genes of representatives of each phylotype were sequenced.

For sequencing, the amplified rRNA gene products were purified using a QIAquick Gel Extraction Spin Kit (QIAGEN, Germany). The purified PCR products were bi-directionally sequenced using forward, reverse and internal primers corresponding to *Escherichia coli* positions 357F (5′-CTCCTACGGGAGGCAGCAG-3′), 926F (5′-AAACTCAAAGGAATTGACGG-3′), 685R (5′-TCTACGCA TTTCACCGCTAC-3′) and 1100R (5′-GGGTTGAGATCGTTG-3′) using Genetic Analyzer ABI 3130XL (Applied Biosystems) with the Big Dye 3.1 terminator protocol. The sequencing reaction was performed with 20 μL reaction mixture containing approximately 50 ng template DNA and 1 pmol of sequencing primers. Post-reaction clean-up and re-suspension was performed for removal of unincorporated dye terminators from the sequencing reaction using 125 mM EDTA, 3M sodium acetate and ethanol. The ethanol-precipitated dried pellet was mixed with 10 μL Hi-Di formamide, heated at 94°C for 2 min for denaturation and quickly chilled on ice. Aliquots (10 μL) of the sample were loaded onto the automated Genetic Analyzer.

### Phylogenetic analyses and diversity indices

The Basic Local Alignment Search Tool (BLAST) (2) programme was used to determine phylogenetic neighbours against the database of type strains with validly published prokaryotic names (21; available online: http://eztaxon-e.ezbiocloud.net/). Molecular Evolutionary Genetics Analysis software (MEGA version 4) was used for phylogenetic analyses (44). The sequences of identified phylogenetic neighbours were aligned with the sequences of the isolates using Clustal W inbuilt with MEGA 4. *Deinococcus radiodurans* (GenBank accession M21413) was used as the outgroup organism. The neighbor-joining method was employed to construct the phylogenetic tree with 1,000 bootstrap replications to assess nodal support in the tree. Based on high similarity (>98.7%) and clear phylogenetic clustering in the same branch, the isolates were assigned to a species.

The number and frequency of phylotypes identified by ARDRA patterns were used to estimate the Shannon diversity index, Simpson’s index, and relative abundance of the three designated sites by using the Paleo-ecology statistics freeware package (PAST) (17). The coverage index (15) as a percentage for each of the sites was assessed to determine how efficiently the sites described the complexity of a theoretical community as an original bacterial community. Various diversity indices were studied to understand the composition (types of bacteria), richness (number of types), and structure (frequency distribution) of the three designated sites.

### Minimal inhibitory concentration (MIC) determination and Uranyl binding

Analytical grade salt of uranyl nitrate, UO_2_(NO_3_)_2_·6H_2_O was used to prepare stock solutions. The stock solutions were sterile filtered through a 0.22 μm nitrocellulose membrane filter (Millipore, India). All the isolates including the type strain *Serratia marcescens* ATCC 13880, *Bacillus lichenoformis* MTCC 429, *Bacillus cereus* MTCC 430, *Arthrobacter ureafaciens* MTCC 3454, *Sphingobacterium multivorum* MTCC 498 and *E. coli* MTCC 118 were grown to the mid-exponential phase in LPM and evaluated for their tolerance to increasing concentrations of U by a factor of two (31). Ten microliters aliquots of mid-exponential phase cultures were spotted onto LPM agar medium plates (150 mm diameter) as described previously (25). The MIC was expressed as the metal concentration that inhibited confluent growth of the spotted culture after 2 d incubation at 28°C (37).

Based on superior tolerance, the uranium binding assay was undertaken for selected representative bacteria from each phylum. The representative isolates along with the type strain *S. marcescens* ATCC 13880 were grown in LPM broth and mid-exponential phase cells were used for uranyl adsorption studies after washing with sterile deionized water. The isolates and the type strain with OD_600_ 2 were resuspended in the U solutions [100 μM (23.8 mg L^−1^) and 2 mM (476 mg L^−1^)] separately and incubated at 30°C under continuous shaking for 24 h. Timed samples (100 μL) were withdrawn and analysed for uranyl binding as described earlier (23).

### Screening of isolates for phosphatase enzyme and metal transporting genes

Histochemical plates were used for detection of phosphatase activity. The identified isolates were either patched or streaked on Luria Bertani (LB) agar plates (pH 7.0) containing 1 mg mL^−1^ phenolphthalein diphosphate (PDP) and 50 μg mL^−1^ methyl green (MG). The plates were incubated at 30°C for 24 h to 48 h. The bacterial phosphatase activity hydrolyses PDP in the medium to phenolphthalein and phosphoric acid and MG (pH indicator) forms intense bluish-green precipitate under acidic conditions, which appears as a zone around the colony. The intensity of the zone was visually monitored over time to screen for phophatase enzyme.

The genomic DNA of the identified Domiasiat isolates was screened for the presence of metal-transporting P_IB_-type ATPase (*zntA/cadA/pbrA-like*) genes. PCR amplification of the P_IB_-type ATPase genes was performed using PCR primers (81JC, 84JC and 133JC) targeted to domain sequences that are found in heavy metal-transporting (P_IB_-type) ATPases (28). PCR mixtures (25 μL) contained approximately 30 ng template DNA, 2 μM forward primer, 2 μM reverse primer, 1.5 mM MgCl_2_ (Taq Buffer), 250 μM each deoxynucleoside triphosphates and 1.0 U Taq polymerase. DNA amplification was carried out in GeneAMP PCR system 9700 with an initial denaturation step of 94°C for 5 min, followed by 30 cycles consisting of denaturation at 94°C for 1 min, annealing at 49°C for 1 min, and extension at 72°C for 1.5 min and then a final extension step of 72°C for 5 min. The expected size of the amplicons was approximately 750 bp.

### Nucleotide sequence accessions numbers

The accession numbers obtained from NCBI Genebank for the 16S rRNA gene sequences (>1,300 bp) are indicated in Table 2 against each isolate.

## Results

### Sampling site description, soil analyses and bacterial enumeration

On the basis of uranium concentration, the sampling points were categorised into three sites (Table 1). Sampling Site I was composed of the sampling points with U concentration ranging from 200 to 1,200 ppm (Fig. S1a). Site II included sampling points with 20–100 ppm U (Fig. S1b), while Site III covered the points with U concentration less than 10 ppm (Fig. S1c). Viable bacterial counts in soil samples of Site I were significantly lower than in the other samples (Table 1), indicating that culturable bacteria decreased in the soil with higher U content.

### Bacterial community analyses

A total of 130 U-tolerant aerobic chemoheterotrophic bacteria (76 Gram-positive and 54 Gram-negative) were isolated using U (1 mM) -enriched LPM plates. Preliminary phenotypic and biochemical characteristics were tested for each isolate, which included colony morphology, oxidative/fermentation test with glucose, motility, catalase, oxidase, and the production of urease, gelatinase, lipase, DNase and few sugar utilization tests (data not shown).

The phenotypic characteristics were supported by the molecular phylogenetic identification approach for characterization of isolates up to species level. The 16S rRNA gene of each of the isolate was amplified and digested with the restriction enzymes *Msp*I and *Hae*III, which resulted in 51 patterns/phylotypes. Isolates showing the same pattern with both enzymes were considered to be representatives of the same species, which was assessed by sequencing the 16S rRNA gene of the representative isolates. To evaluate the phylogenetic diversity represented by 51 ARDRA patterns, one or more representative isolates of the most predominant ARDRA patterns were sequenced. Sixty-two of the 130 isolates were identified up to species level based on high similarity (>98.7%) and clear phylogenetic clustering in the same branch.

Among the identified U-tolerant bacteria, Gram-positive bacteria comprised *Firmicutes* and *Actinobacteria* while Gram-negative bacteria isolates included *Betaproteobacteria*, *Gammaproteobacteria* and *Bacteroidetes*. Among these 5 phyla, *Firmicutes* were obtained from all the sampling sites but with high abundance at Site I and III. *Actinobacteria* were obtained only from Site I, which incidentally had the highest U concentration, while *Bacteroidetes* were not found at site III, which had the lowest U concentration. *Gammaproteobacteria* were the most abundant phyla from Site II, with *Burkholderia* being the only *Betaproteobacteria* (Table 2).

Among the *Firmicutes*, the most abundant isolates were associated with genus *Paenibacillus* (13 analysed sequences corresponding to nine ARDRA patterns from 24 isolates) and *Bacillus* (11 sequences corresponding to 10 ARDRA patterns from 22 isolates). *Arthrobacter* (four sequences corresponding to four ARDRA patterns from seven isolates) and *Microbacterium* (two sequences corresponding to two phylotypes from four isolates), were the most abundant *Actinobacteria. Betaproteobacteria*, was solely represented by *Burkholderia arboris* (one sequence analysed from one phylotype among two isolates). *Serratia* (nine sequences corresponding to six ARDRA patterns from 21 isolates) and *Pseudomonas* (three sequences corresponding to three phylotypes from seven isolates) were the most abundant *Gammaproteobacteria. Sphingobacterium* (five sequences corresponding to three phylotype from 16 isolates) was the most abundant genus in the phylum *Bacteroidetes*.

Sequences of more than 1,300 bp length were used to perform BLAST search against the database of type strains at EzTaxon-e identification service to identify the nearest phylogenetic neighbours. The isolates could be affiliated up to species level, by their high sequence similarity (>98.7%) against the database of type strains (42, 43) with validly published prokaryotic names (Table 2) and by high phylo-genetic relatedness between the studied strains and the closest type strain (Fig. S2a, b, c). Simulation studies suggests that under favourable conditions (roughly equal rates of change, symmetric branches), bootstrap values greater than 70% correspond to a probability of greater than 95% of phylogeny being found (18), which is validated by molecular phylogenetic analysis of the isolates in the present study. Hence, 59 of the isolated U-tolerant bacteria were identified up to species level, leaving three *Paenibacillus* species isolates, KMS2U3, KMS2U5 and NONG6, which displayed lower than 98.7% pairwise sequence similarity with the closest species.

The diversity indices in the present study do not depict the overall diversity as the isolation was conditioned on enrichment medium. The maximum diversity of U-tolerant bacteria as evident by the Shannon value, evenness and Simpson’s indices was observed at Site II, which had the intermediate concentration of U (Table 3). Coverage percentage (the percentage of isolates that were at least duplicated in the phylotype) indicated the minimum presence of U-tolerant bacteria at Site III, which had the lowest U concentration.

### Tolerance and bioadsorption of U, profile of phosphatase enzyme and metal transporting genes in bacterial isolates

Out of the total identified Domiasiat natural isolates, 90.32% showed MIC of 4.0 mM for U as compared to MIC of 2.0 mM shown by type strains *S. marcescens* ATCC 13880, *B. lichenoformis* MTCC 429, *B. cereus* MTCC 430, *S. multivorum* MTCC 498 and *E. coli* MTCC 118 and MIC of 1.0 mM was shown by type strain *A. ureafaciens* MTCC 3454 (Table 2). There was no precipitation in the used minimal medium, making the metal uniformly available in the medium.

**Fig. 1:**
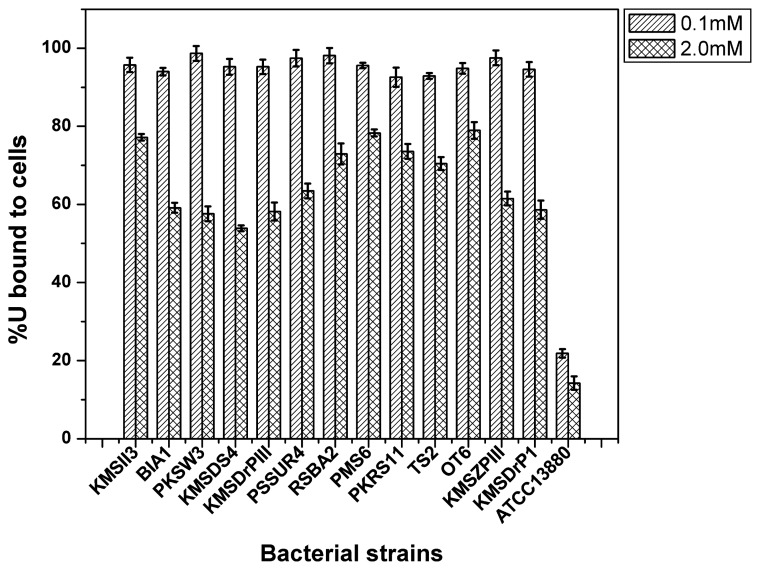
Uranium (U) binding by representative isolates and type strain *S. marcescens* ATCC 13880. Equivalent cells at OD_600_ 2 were exposed to 100 μM and 2 mM uranyl nitrate solutions and were assayed for bound U. U was estimated in the cell pellets following 24 h incubation. (Error bars represent ±5% of the value.)

The Domiasiat isolates tolerating toxic concentrations of soluble U(VI), were assessed for their uranyl adsorption properties. Isolates which tolerated 2 mM U were randomly chosen for the uranyl bioadsorption assay. In our earlier report (23), we observed that U adsorption was highest at a low concentration of 100–500 μM and the efficiency of U adsorption decreased with increasing U concentration and, at higher concentration (>1 mM), the adsorption capacity was lowest among five *S. marcescens* species. In this study, U scavenging was observed at two concentrations of U, *i.e.* the lowest at 100 μM and highest at 2 mM. In agreement with an earlier study, uranyl binding by all these isolates was also found to be very rapid and monophasic, reaching equilibrium by 1 h, and was maintained until 24 h. It was observed that 13 representative strains were able to remove about 90–98% (21–23 mg L^−1^) and 53–78% (250–371 mg L^−1^) of U(VI) on being challenged with 100 μM and 2 mM uranyl nitrate solutions, respectively, at pH 3.5 within 10 min of exposure and the activity was maintained until 24 h of incubation (Fig. 1). The type strain used could only bind 15–20% U from the test solutions (100 μM or 2 mM) at pH 3.5. U remained stable under our experimental conditions and did not precipitate, as seen in the abiotic controls. Uranyl adsorption was not evaluated beyond 24 h as there was a decrease in cell density due to nutrient limitation. The natural U-tolerant Domiasiat isolates indicated effective U bioadsorption.

The natural isolates were screened for phosphatase activity by visually observing for intense bluish-green methyl green precipitation zone in and around the colonies in histochemical plates (Fig. S3). Out of the 62 analysed bacteria, 47 isolates showed phosphatase activity (Table 2).

The amplification of approximately 750 bp of the metal-transporting gene was obtained from 33 isolates (22 Gram-positive and 10 Gram-negative bacteria) from the 62 isolates. Sixteen representative isolates (Table S1) of different phyla were selected for sequencing and further analysis. The degenerative primers were targeted for known sequences of *zntA*/*cadA*/*pbrA*-like genes, which are specific to genus *Bacillus*, *Acinetobacter*, *Pseudomonas* and *Arthrobacter* (7, 28); however, we succeeded in obtaining the expected amplicons from other genus as well, such as *Lysinibacillus*, *Paenibacillus*, *Chryseobacterium* and *Sphingobacterium* using the earlier reported primers (7, 28). Martinez *et al.* (28) reported amplification of the *copA*-like gene in only two Gram-negative strains among 50 randomly chosen P_IB_-type ATPase-positive lead-resistant strains instead of the expected *zntA*/*cadA*/*pbrA*-like genes using the same primer set. In the present study, we observed the expected PCR amplicons for *zntA*/*cadA*/*pbrA* loci from 10 Gram-negative bacteria and sequence analysis revealed *zntA*-related genes from five, isolates *viz.*, PMSZPI, KMSDrP1, KMSDrP2, KMSDrP3 and KMSZPIII, all belonging to the phylum *Bacteroidetes*, while isolate OT6 under phylum *Gammaproteobacteria* was identified with the *copA*-related gene (Table S1). An unexpected *copA*-related gene was observed from a Gram-positive bacterium RSBA2 belonging to the phylum *Actinobacteria*.

## Discussion

The present study was the first attempt to study the culturable U-tolerant bacterial diversity from the premined U ore deposit in Domiasiat, India. The soil samples analysed from a geochemically unique habitat of exploratory drilling and test recovery plant showed acidic pH (4.3–6.3), which is attributed to the known presence of pyrite (FeS_2_) and humic substance (coal) in the prevailing sandstone rocks (24). The soil samples had a moderate percentage of organic carbon but uranium concentration ranged from <10 ppm to 1,200 ppm. U mineralization was said to be confined to grey pyritic sandstone with intercalated carbonaceous seams at a depth of 40–45 m (11); however, surface U anomalies occurred across the dissected Kylleng Nala section (8), and this could be the reason for certain sampling points showing a very high U presence across the section as compared to other sub-surface soil having comparatively low U occurrence. Apart from the surface U anomaly across the dissected Kylleng Nala section, the reason for the varied U occurrences could be attributed to the chemical property of U of being highly leachable. Other metal concentrations were found to be within the range of the background concentration of trace elements, as observed in non-anthropogenic soils (4).

Low-nutrient medium such as R2A agar was used for determining the bacterial biomass, which when incubated for longer incubation periods allowed the nutritionally stressed bacteria to grow well. The low counts of aerobic heterotrophs from the high U soil of Site I was in agreement with reports from heavy metal- and radionuclide-contaminated sites at Hanford (12) and at the Field Research Center (FRC) in Oak Ridge, TN (28). The bacterial counts from Site II remained one to two orders of magnitude higher than Site I while Site III showed the highest counts, exceeding Site I by four orders of magnitude, corroborating well with the low U presence (<10 ppm).

Based on the molecular analysis, 16 bacterial genera comprising 34 different species were identified using the nucleotide sequence database from EzTaxon-e Identification Service, which has a manually curated database of type strains of prokaryotes and provides authentic identification tools using a similarity-based search. Three of the reported isolates, KMS2U3, KMS2U5 and NONG6, were indicative of novel species as 1,461, 1,379 and 1,369 bp of 16S rRNA gene sequences, respectively, showed lower than 98.7% similarity with their closest matches. Since 16S rRNA gene sequence similarity is considered for delineating novel taxa and in the identification of isolates (6), the possible occurrence of novel species cannot be ruled out and such a finding suggests that the site is geochemically distinct, harbouring novel bacteria. Previously, the proposed cut-off value for the delineation of prokaryotic species that is well accepted among microbiologists was 97% (42); however, recently, this cut-off value has been made more specific, 98.7–99%, after inspection of a large amount of recently published data (43). Heavy metals and radionuclides have played major role in limiting microbial diversity in several such environments (30); however, in the present study, regardless of high U presence (Site I and II), a high diversity index was observed which could possibly be attributed to the isolation methodology, which specifically targeted the diversity of U-tolerant bacteria, and to the availability of a moderate percentage of organic carbon in the soil samples (19).

In spite of the consistency in the selection of growth media and conditions, there were significant variations in the obtained phyla of bacteria from different sampling sites. The most abundant bacterial phylum obtained from the sites was *Firmicutes* and this phylum is reported to be present in a number of metal/radionuclide-contaminated soils (36) and is a predominant phylum in the sediments in which U(VI) was being adsorbed (32). The distribution of *Actinobacteria*, *Bacteroidetes*, *Gammaproteobacteria* corroborates with reports from radionuclide- and heavy metal-contaminated subsurface soils at the FRC (28), U mine complex (SD) (36), U depository site in Gunnison, CO, from U mill tailings in Shiprock, NM (34) and from an U ore sample from Jaduguda U mine, India (19).

Earlier studies have suggested that natural microorganisms adopt various strategies to adapt to heavy metal-rich environments by employing mechanisms such as biosorption, bioprecipitation, extracellular sequestration, transport, and/or chelation, which form the basis of the utilisation of these organisms for various bioremediation approaches (16). The MIC study performed in the present study clearly indicated the higher tolerance capacity of the natural isolates as compared to the type strains examined under the same laboratory conditions. Based on superior U resistance, the Domiasiat isolates were tested for U binding. Similarly, a U binding study from a groundwater sample of a radioactive repository from 200 μM solution of U at pH 5 showed removal ability ranging between 21% and 90% of the added U, differing between the studied strains (14). Our study used acidic pH, characteristic of most U waste, and tested the binding capacity at both low and high U concentrations. At higher concentrations (2 mM), the lower binding capacity (53–78%) of all the isolates may be attributed to the saturation of binding sites on the cell surfaces. Although the binding capacity was observed to be very low, two Gram-positive bacteria *viz.*, *B. cereus* KMSII3 (77%) and *Arthrobacter mysorens* RSBA2 (73%), and three Gram-negative bacteria *viz.*, *Burkholderia arbores* PMS6 (78%), *Pseudomonas ficuserectae* PKRS11 (73.5%) *Pseudomonas koreensis* OT6 (79%), were observed to be the most promising isolates for U sequestration. At a low concentration of U, although all the tested isolates showed removal of above 92% of added U, the most efficient among them were three Gram-positive bacteria *viz.*, *Microbacterium azadirachtae* PSSUR4 (97%), *A. mysorens* RSBA2 (98%), *Bacillus isronensis* PKSW3 (98.5%), and one Gram-negative bacteria *viz.*, *Sphingobacterium siyangense* KMSZPIII (97.5%).

Earlier reports have suggested that metals can be precipitated *via* enzymatically generated ligands such as sulphide or phosphate (5, 27) and enzymatic bioprecipitation of heavy metals as metal phosphates in particular is very attractive since it can recover metals from very low concentrations not amenable to chemical techniques (27). We observed that 76% of our natural isolates possessed constitutive phosphatase activity, of which 12% have intracellular phosphatase while the remaining 88% possess both intra- as well as extracellular phosphatase activity. Evidence of U(VI) precipitation *via* the phosphatase activity of naturally occurring isolates from radio-nuclide and metal-contaminated subsurface soils, playing an important role in the bioremediation of U, was reported previously (3, 29). Similarly, the phosphatase liberation potential of these natural isolates could be utilised for the bioremediation activity of U using a precipitation mechanism.

The ability of natural isolates to survive under such conditions also prompted us to look at one of the basic defence mechanisms against high metal concentration known to be utilised by heavy metal-transporting transmembrane proteins. Amplification of *zntA*/*cadA*/*prbA*-specific ATPase genes from a genus other than the targeted genera and the *copA*-like gene instead of the expected *zntA*/*cadA*/*pbrA* loci were also observed. Such unexpected observations were theorized to be possible, considering the limited number of control strains tested using the initial primer sets for the P_IB_-type *zntA*/*cadA*/*prbA*-specific ATPases (7, 28). In present study, we observed that only seven isolates, *viz.*, WAKH2, NONG2, LONG1, KMSDS4, RSBA2, PMSZPI and KMSII3, showed similarity (>80%) to the closest P_IB_-type ATPase sequence of the same genus identified using the 16S rRNA gene in the GenBank database, while the rest showed similarity to another genus or to the same genus but with very poor similarity (Table S1). This suggests the possible horizontal transfer of this gene, corroborating a report on subsurface bacteria from metal-contaminated sites (28).

The identification and documentation of natural U-tolerant bacteria from the ecologically distinct habitat of a U ore deposit in Domiasiat, India can be regarded as baseline information on cultivated pure indigenous U-tolerant bacterial communities, which could possibly serve as an indicator to gauge the degree of environmental impact likely to be caused by the proposed mining in the near future. This study analysed the natural isolates for properties such as U tolerance, binding of U, presence of phosphatase enzyme and metal-transporting genes, supporting the relevance of the natural isolates as vital candidates for bioremediation of U-contaminated wastes.

## Supplementary Material



## Figures and Tables

**Table 1:** Geo-microbial properties of soil samples from different sampling sites

	Site I			Site II			Site III			
			
	PB1	KMS1	PMS	KMS1A2TS	KMS1DrP	BIA	KMS1A2OT	PV	LV	WJ
GPS	N25°19.337′ E91°12.354′	N25°19.285′ E91°12.594′	N25°19.140′ E91°12.705′	N25°19.121′ E91°12.486′	N25°19.271′ E91°12.599′	N25°19.251′ E91°12.341′	N25°19.092′ E91°12.511′	N25°19.059′ E91°12.185′	N25°20.360′ E91°11.764′	N25°21.649′ E91°15.737′
Altitude (m)	766	824	814	826	815	800	818	801	852	1304
pH	5.82	5.655	5.36	5.3	5.11	5.25	5.11	4.75	5.3	4.53
OC^a^ (g kg^−1^)	5.1–6.0	7.5–8.0	7.5–8.0	1.0–3.0	7.5–8.0	7.5–8.0	8.0–10.0	8.0–10.0	8.0–10.0	8.0–10.0
Elements (mg kg^−1^)										
Cu	16	28.8	16	7.8	7.3	10.1	10.2	14.8	24.8	24.3
Pb	20	23	22	12	11	11	10	10	9	13
Zn	344.3	377	165.3	134.9	115.2	194.9	194.9	245.7	412.5	200.5
Cd	1.7	0.9	2.7	1.5	2.7	2.6	2.6	1.2	3.8	2.3
U	1200	480	200	70	100	20	<10	<10	<10	<10
Microbial counts (CFU/gm)	1.01E+4	7.26E+4	5.36E+4	1.80E+5	1.90E+6	2.30E+6	1.20E+8	2.19E+8	1.48E+8	2.46E+8

a OC: Organic carbon available in soil

**Table 2:** Identification of the isolates based on the percentage similarity of the 16S rRNA genes with validly published strains names in the Eztaxon database, and representative phylotypes showing the site distribution of the abundance of bacterial isolates; screening of phosphatase activity using histochemical plates and profiling of metal-transporting P_IB_-type ATPase genes

Closest Match	% sim^a^	Sequenced Isolates	Accession Number	nt^b^	Area with No. of Isolates^c^			Phylotype	MIC of U	Phosphatase	P_IB_-type ATPase

					Site I	Site II	Site III				
***Firmicutes***											
*Bacillus licheniformis*	99.79	OT3	HQ232299	1428			2	1	4.0	+	+
*Bacillus altitudinis*	99.8	PMSI	GQ468396	1506		1		1	4.0	+	+
*Bacillus cereus*	100	KMSII3	GQ468395	1459		3		1	4.0	−	+
*Bacillus halmapalus*	99.08	LONG2	JN164001	1411			1	1	4.0	+	+
*Bacillus thuringiensis*	>99.8	KMSZP5	JF768711	1424	2				4.0	−	+
		PKSWII	JF768713	1467	3				2.0	+	+
		PSSUIII	JF768715	1457	3			4	2.0	+	+
		KMSDrPIII	JF768710	1433		2			4.0	+	+
		NONG4	JN164004	1400			3		4.0	+	+
*Bacillus isronensis*	99.59	PKSW3	JF768712	1446	2			1	4.0	−	+
*Bacillus pseudomycoides*	99.93	PKSWIII	JF768714	1458	2			1	2.0	+^e^	−
*Lysinibacillus xylanilyticus*	>99.6	KMSDS4	JF768718	1463					4.0	−	+
		LONG1	JN164000	1425		3	3	3	4.0	+	+
		NONG2	JN164003	1456			2		4.0	+	+
		WAKH2	JN164007	1397			2		4.0	+	+
*Lysinibacillus fusiformis*	100	KMSDS3	JF768717	1460		2		1	4.0	+	−
*Lysinibacillus sphaericus*	100	LONG4	JN164002	1403			1	1	4.0	+	−
*Staphylococcus arlettae*	99.93	KMSZP2	JN230423	1455	1			1	4.0	+^e^	−
*Staphylococcus warneri*	99.93	KITS2	GU270571	1460		2		1	2.0	−	+
*Paenibacillus taichungensis*^d^	98.69	KMS2U3	JF768722	1461			1		4.0	+	−
*Paenibacillus nanensis*^d^	97.90	KMS2U5	JF768731	1379			1		4.0	−	−
*Paenibacillus amylolyticus*^d^	98.53	NONG6	JN164005	1369			1		4.0	−	−
*Paenibacillus alkaliterrae*	98.71	WAKH1	JN164006	1401			1		4.0	+^e^	−
*Paenibacillus taichungensis*	98.82	BIA1	JF768721	1455		1			4.0	+	−
*Paenibacillus taichungensis*	>99.4	PKSW2	JF768725	1466	2			9	4.0	+^e^	−
		PSSUR2	JF768729	1465	3				4.0	−	+
		PSSUR1	JF768728	1459	2	3			4.0	−	−
		PSSUR3	JF768730	1462	2				4.0	−	+
		PKSW1	JF768724	1462	3				4.0	−	+
		PMSZP4	JF768726	1457					4.0	−	−
*Paenibacillus pabuli*	>99.3	PSS2	JF768727	1469	2	2			4.0	−	+
		KMSDS2	JF768723	1500					4.0	−	+
***Actinobacteria***											
*Arthrobacter ureafaciens*	99.36	KMSZP1	JF768706	1408	1				2.0	+	−
*Arthrobacter mysorens*	99.02	RSBA2	JF768709	1436	2			4	4.0	+^e^	+
*Arthrobacter nicotinovorans*	>99.20	RSBA1	JF768708	1428	2				4.0	+	−
		KMSZP6	JF768707	1435	2				4.0	+	+
*Microbacterium azadirachtae*	99.34	PSSUR4	JF768719	1431	2			1	4.0	+	+
*Microbacterium aerolatum*	99.70	RSBA5	JF768720	1419	2			1	4.0	+	−
*Rhodococcus equi*	99.37	PSS3	JF768732	1429	1			1	4.0	+^e^	−
***β-Proteobacteria***											
*Burkholderia arbores*	99.86	PMS6	GQ468397	1440		2		1	4.0	+	−
***γ-Proteobacteria***											
*Stenotrophomonas maltophilia*	99.30	KITS1	GU270570	1442		2		1	2.0	+	+
*Pseudomonas koreensis*	>99.4	OT6	HM747953	1413			2	3	4.0	+	+
		TS2	HM747951	1416			3		4.0	+	+
*Pseudomonas ficuserectae*	98.86	PKRS11	HM747952	1405		2			4.0	+	−
*Enterobacter kobei*	99.21	TS4	HQ232300	1399			2	1	4.0	+	−
*Citrobacter freundii*	99.79	KMSI3	GQ468398	1410		2		1	4.0	+	+
*Acinetobacter beijerinckii*	98.82	KITS3	GU270572	1439		2		1	4.0	−	−
*Serratia marcescens* subsp. *Sakuensis*	>99.5	PKRS1	GU270569	1494		2			4.0	+	−
		PKRS2	GQ468401	1481		3		6	4.0	+	−
		KMS9	GU270568	1485		3			4.0	+	−
		OTII7	GQ468400	1482			2		4.0	+	−
*Serratia marcescens* subsp. *Marcescens*	>99.5	KMS4	GU270567	1439		2			4.0	+	−
		PKRS5	HM747950	1410		3			4.0	+	−
		TS1	HM747949	1443			1		4.0	+	−
		OT4	HM747954	1439			2		4.0	+	−
		PMS1	HM747955	1441		3			4.0	+	−
***Bacteroidetes***											
*Chryseobacterium culicis*	98.87	PMSZPI	JF768716	1420		3		1	4.0	+	+
*Sphingobacterium siyangense*	>98.95	KMSDrP1	JF768733	1440		2			4.0	+	+
		KMSDrP2	JF768734	1414		3			4.0	+	+
		KMSDrP3	JF768737	1438		2		3	4.0	+	+
		KMSZPIII	JF768735	1445	2				4.0	+	+
		PSSUR5	JF768736	1343	4				4.0	+	+

a Similarity percentage with recognised type strain of validly published prokaryotic names (available online http://eztaxon-e.ezbiocloud.net/)

b Length of 16S rRNA gene sequence

c Isolates selected based on unique colony morphology

d Low similarity percentage with the closest match, may not be appropriately affiliated to species level

e Only intracellular phosphatase activity (+: Positive result; −: Negative result)

**Table 3:** Diversity of U-tolerant bacterial communities at the three designated sites based on ARDRA patterns (phylotypes)

Site	Isolates^a^ (*N*)	Richness^b^ (*S*)	Coverage^c^ (C)	Shannon^d^ (*H*)	Evenness^e^ (*E*)	Simpson^f^ (*D*)
I	45	21	93 (3)	2.99	0.947	0.947
II	55	24	96 (2)	3.14	0.963	0.955
III	30	17	80 (6)	2.75	0.918	0.931

a *N*: Number of individuals that were grouped by ARDRA patterns

b *S*: Number of distinct phylotypes based on ARDRA patterns

c C (Coverage index) = 1 −(n/*N*), where n is the number of phylotypes appearing only once (values in parentheses represent ‘n’, also termed as singletons)

d *H* (Shannon index) = −∑*p*_i_ ln *p*_i_, where *p*_i_ is the ratio of isolates of each phylotype

e *E* (Evenness index) = H/lnS

f *D* (Simpson’s index) = *p*_i_^2^
