# Supplementary material for: Uranium (U)-Tolerant Bacterial Diversity from U Ore Deposit of Domiasiat in North-East India and Its Prospective Utilisation in Bioremediation
Source: Microbes Environ. 2012 Oct 19;28(1):33–41. doi: 10.1264/jsme2.ME12074 (PMC4070695; doi:10.1264/jsme2.ME12074)
Supplement: Supplementary file 1 [file 28_33_s1.pdf]

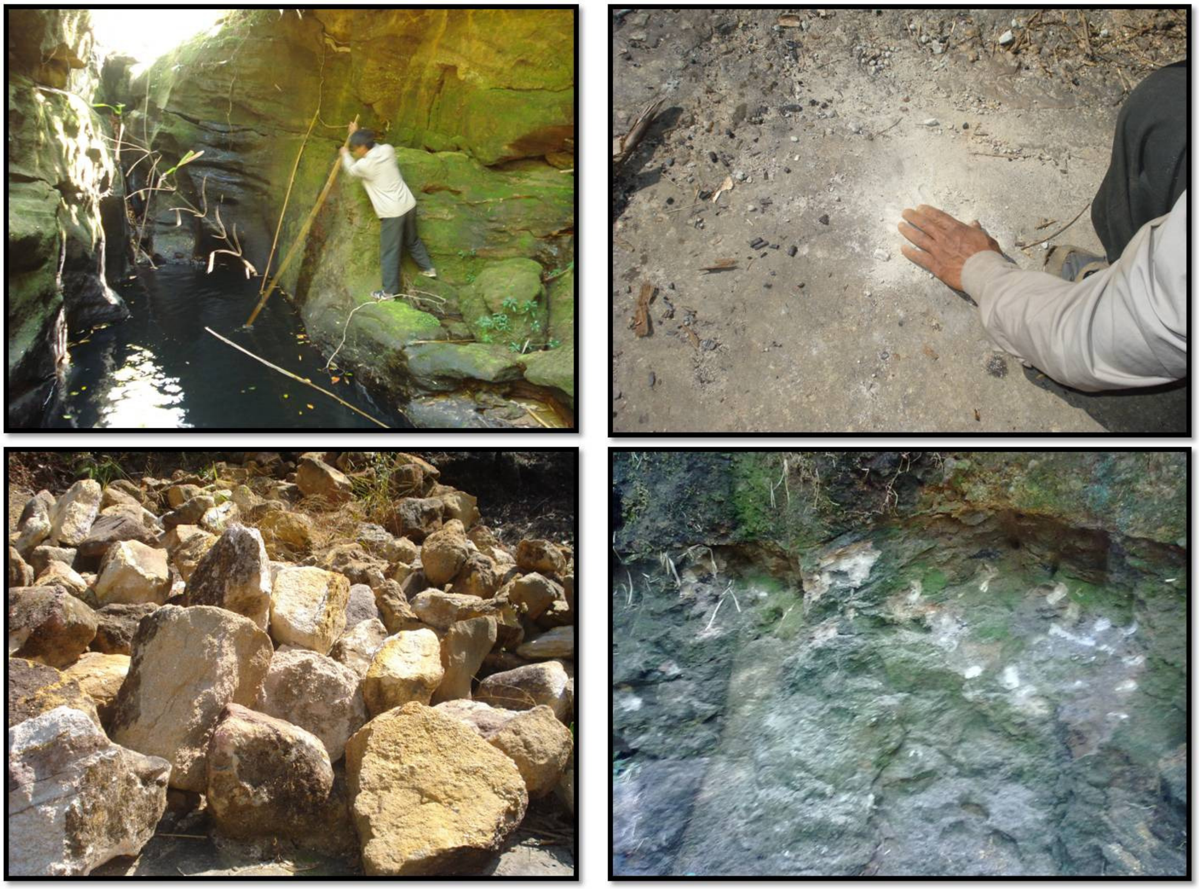

S1(a)

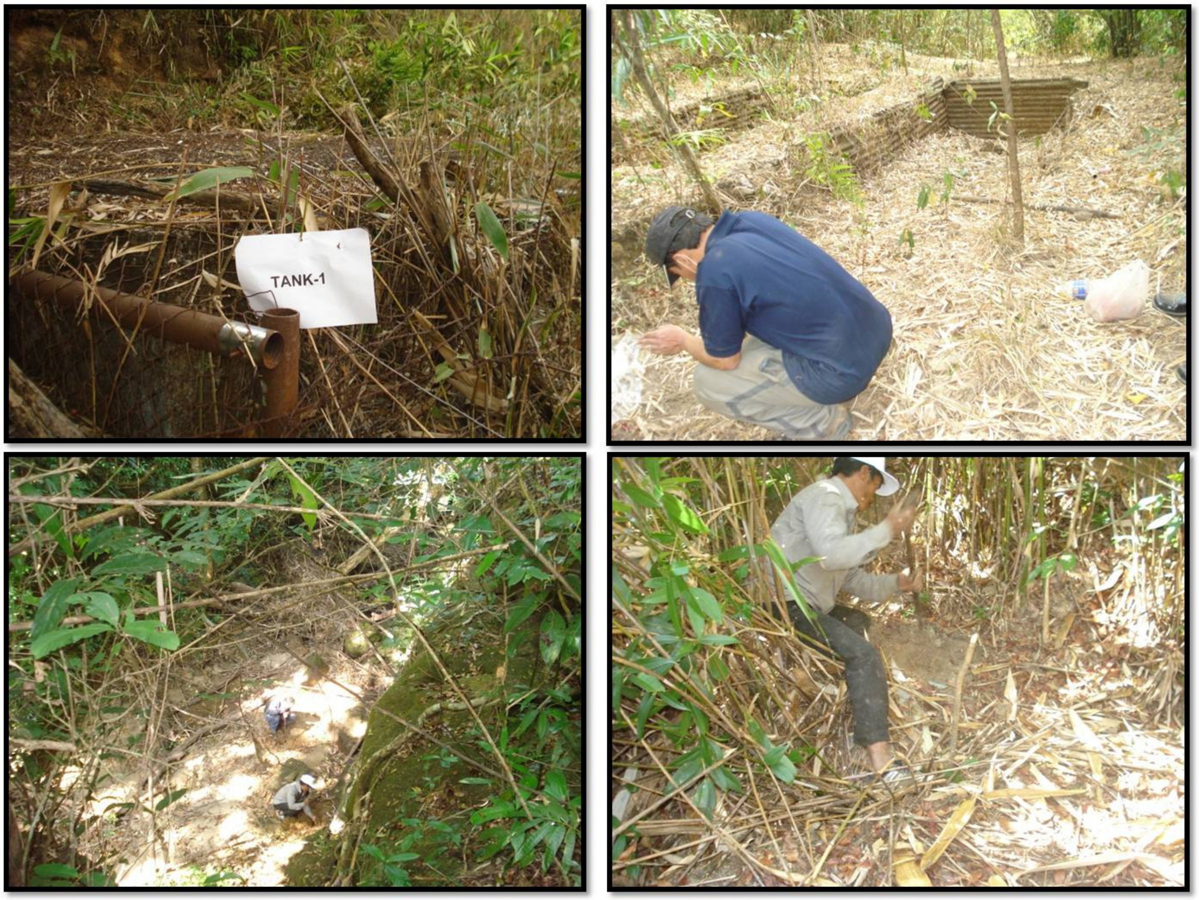

S1(b)

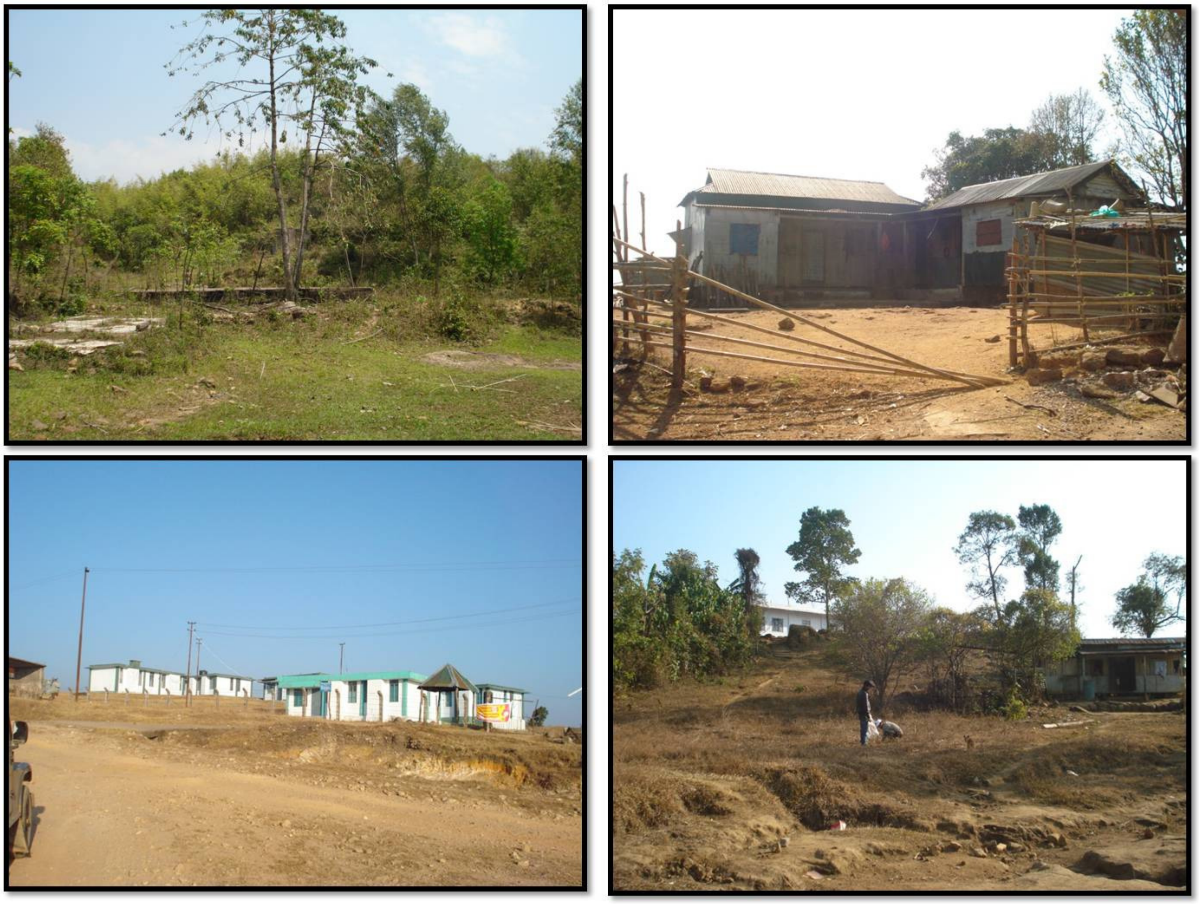

S1(c)

Fig. S1 Sampling site description. (a) Snapshots of sampling Site I showing Kylleng Nala and the greyish-white rocky surface across dissected Nala section, (b) Snapshots of sampling Site II which lies around the designated mining site, (c) Snapshots of sampling Site III which comprises of sample around mining site and from adjoining areas

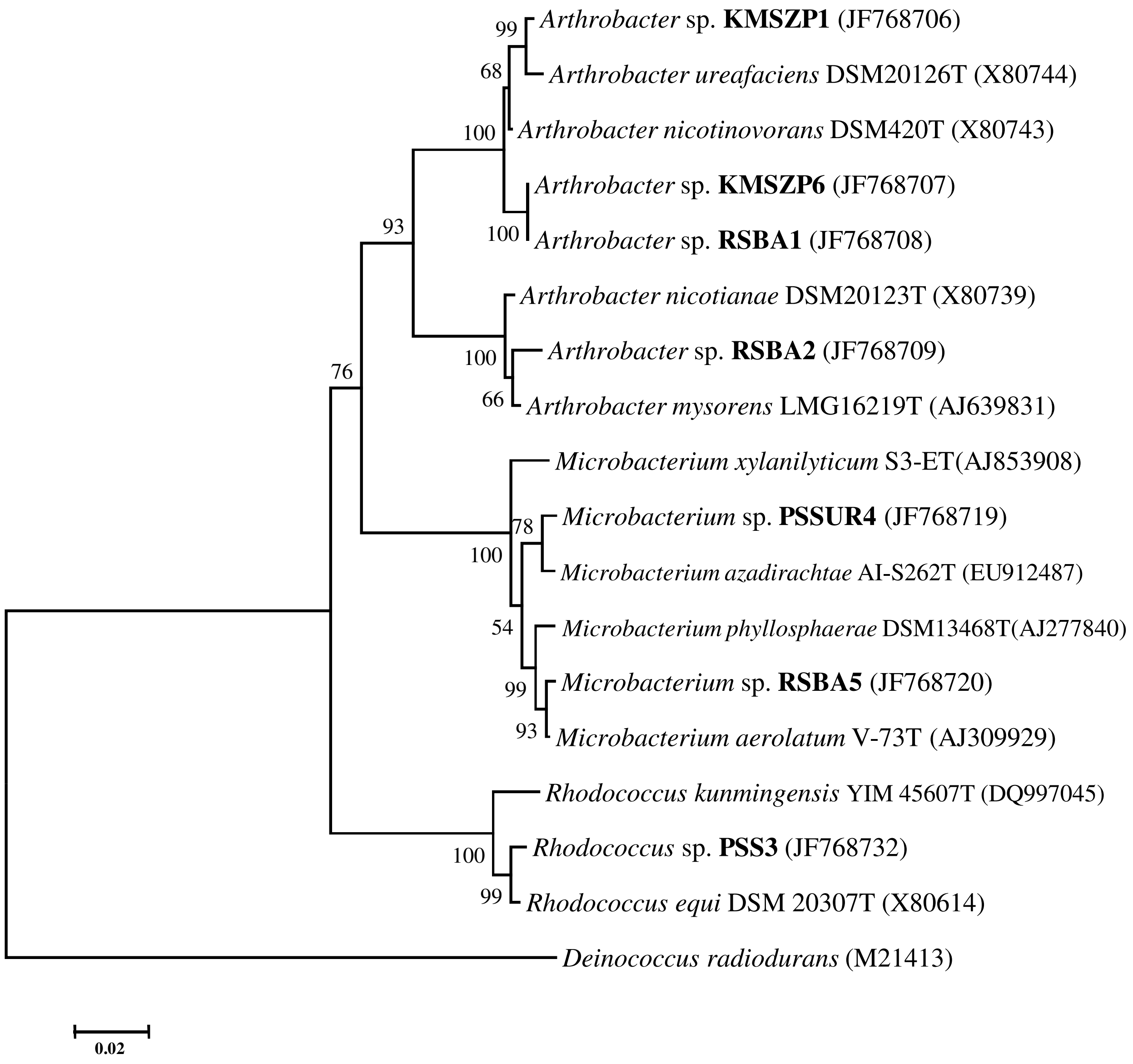

99
68
100
93
100
100
66
76
78
100
54
99
93
100
99
Arthrobacter sp. KMSZP1 (JF768706)
Arthrobacter ureafaciens DSM20126T (X80744)
Arthrobacter nicotinovorans DSM420T (X80743)
Arthrobacter sp. KMSZP6 (JF768707)
Arthrobacter sp. RSBA1 (JF768708)
Arthrobacter nicotianae DSM20123T (X80739)
Arthrobacter sp. RSBA2 (JF768709)
Arthrobacter mysorens LMG16219T (AJ639831)
Microbacterium xylanilyticum S3-ET(AJ853908)
Microbacterium sp. PSSUR4 (JF768719)
Microbacterium azadirachtae AI-S262T (EU912487)
Microbacterium phyllosphaerae DSM13468T(AJ277840)
Microbacterium sp. RSBA5 (JF768720)
Microbacterium aerolatum V-73T (AJ309929)
Rhodococcus kunmingensis YIM 45607T (DQ997045)
Rhodococcus sp. PSS3 (JF768732)
Rhodococcus equi DSM 20307T (X80614)
Deinococcus radiodurans (M21413)
0.02

S2(a)

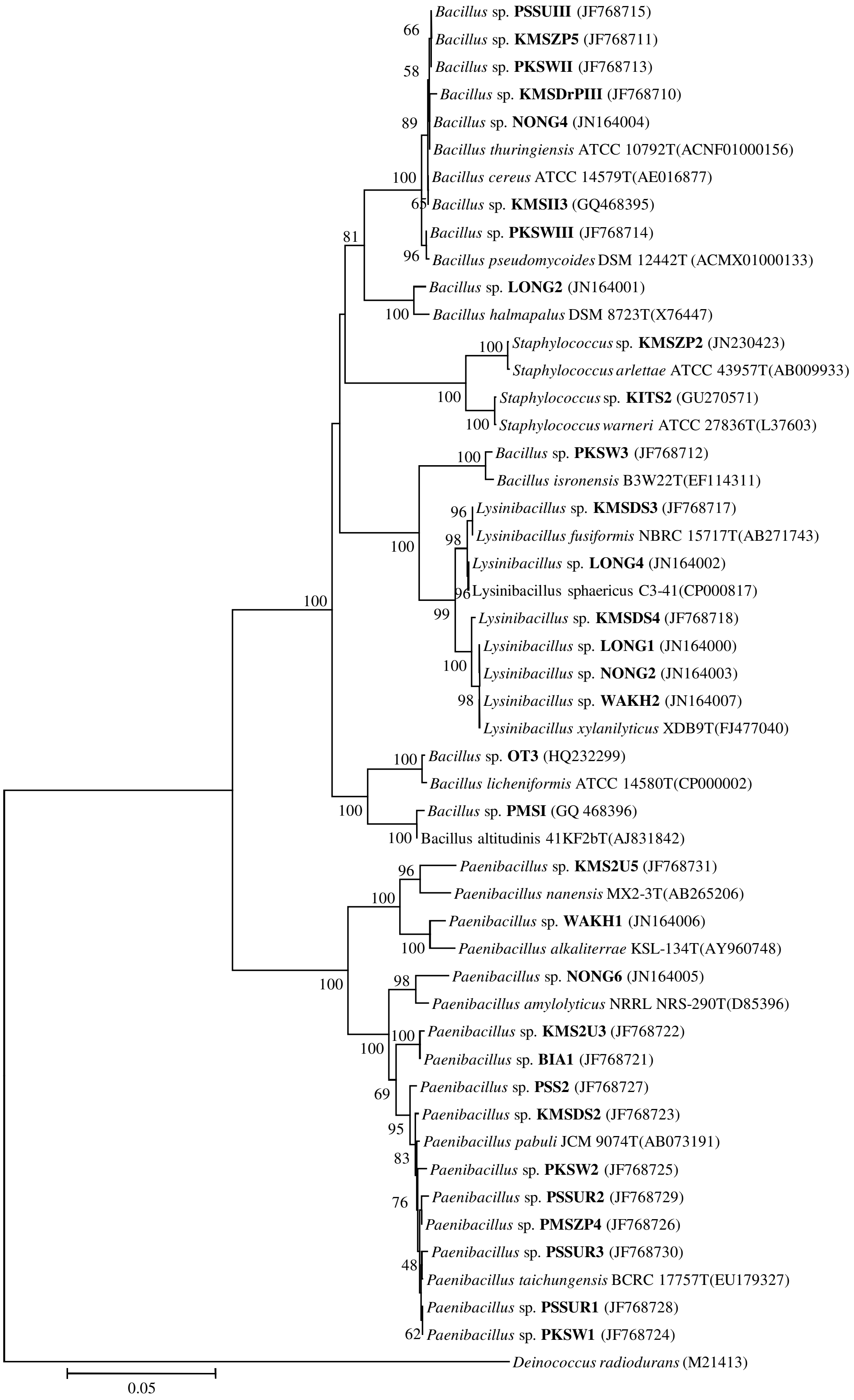
Bacillus sp. PSSUIII (JF768715)
Bacillus sp. KMSZP5 (JF768711)
Bacillus sp. PKSWII (JF768713)
Bacillus sp. KMSDrPIII (JF768710)
Bacillus sp. NONG4 (JN164004)
Bacillus thuringiensis ATCC 10792T(ACNF01000156)
Bacillus cereus ATCC 14579T(AE016877)
Bacillus sp. KMSII3 (GQ468395)
Bacillus sp. PKSWIII (JF768714)
Bacillus pseudomycoides DSM 12442T (ACMX01000133)
Bacillus sp. LONG2 (JN164001)
Bacillus halmapalus DSM 8723T(X76447)
Staphylococcus sp. KMSZP2 (JN230423)
Staphylococcus arlettae ATCC 43957T(AB009933)
Staphylococcus sp. KITS2 (GU270571)
Staphylococcus warneri ATCC 27836T(L37603)
Bacillus sp. PKSW3 (JF768712)
Bacillus isronensis B3W22T(EF114311)
Lysinibacillus sp. KMSDS3 (JF768717)
Lysinibacillus fusiformis NBRC 15717T(AB271743)
Lysinibacillus sp. LONG4 (JN164002)
Lysinibacillus sphaericus C3-41(CP000817)
Lysinibacillus sp. KMSDS4 (JF768718)
Lysinibacillus sp. LONG1 (JN164000)
Lysinibacillus sp. NONG2 (JN164003)
Lysinibacillus sp. WAKH2 (JN164007)
Lysinibacillus xylanilyticus XDB9T(FJ477040)
Bacillus sp. OT3 (HQ232299)
Bacillus licheniformis ATCC 14580T(CP000002)
Bacillus sp. PMSI (GQ 468396)
Bacillus altitudinis 41KF2bT(AJ831842)
Paenibacillus sp. KMS2U5 (JF768731)
Paenibacillus nanensis MX2-3T(AB265206)
Paenibacillus sp. WAKH1 (JN164006)
Paenibacillus alkaliterrae KSL-134T(AY960748)
Paenibacillus sp. NONG6 (JN164005)
Paenibacillus amylolyticus NRRL NRS-290T(D85396)
Paenibacillus sp. KMS2U3 (JF768722)
Paenibacillus sp. BIA1 (JF768721)
Paenibacillus sp. PSS2 (JF768727)
Paenibacillus sp. KMSDS2 (JF768723)
Paenibacillus pabuli JCM 9074T(AB073191)
Paenibacillus sp. PKSW2 (JF768725)
Paenibacillus sp. PSSUR2 (JF768729)
Paenibacillus sp. PMSZP4 (JF768726)
Paenibacillus sp. PSSUR3 (JF768730)
Paenibacillus taichungensis BCRC 17757T(EU179327)
Paenibacillus sp. PSSUR1 (JF768728)
Paenibacillus sp. PKSW1 (JF768724)
Deinococcus radiodurans (M21413)
66
58
89
100
65
96
81
100
100
100
100
100
100
96
98
96
99
100
98
100
100
100
100
96
100
100
98
100
100
100
69
95
83
76
48
62
0.05

S2(b)

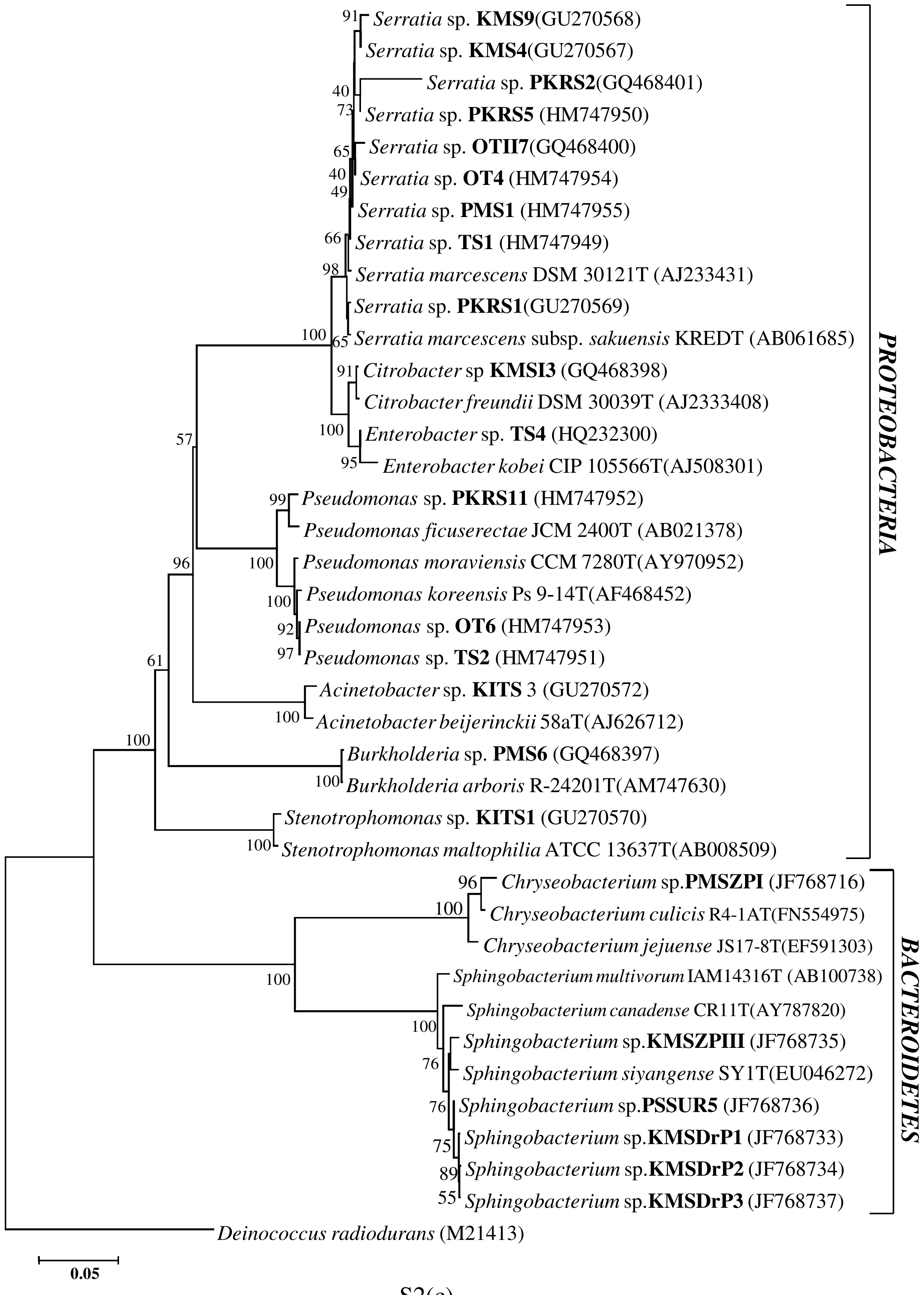

S2(c)

Fig. S2 Neighbor-joining tree based on 16S rRNA (more than 1300 bases) gene sequences representing the phylogenetic relationships between the representative (a) high GC, gram positive (Firmicutes) ; (b) low GC, gram positive (Actinobacteria) ; (c) proteobacteria and bacteroidetes; and the related species obtained from the database of type strains with validly published prokaryotic names at EzTaxon-e identification service. *Deinococcus radiodurans* (M21413) was taken as the outgroup organism. The scale bar corresponds to the expected number of changes per nucleotide position.

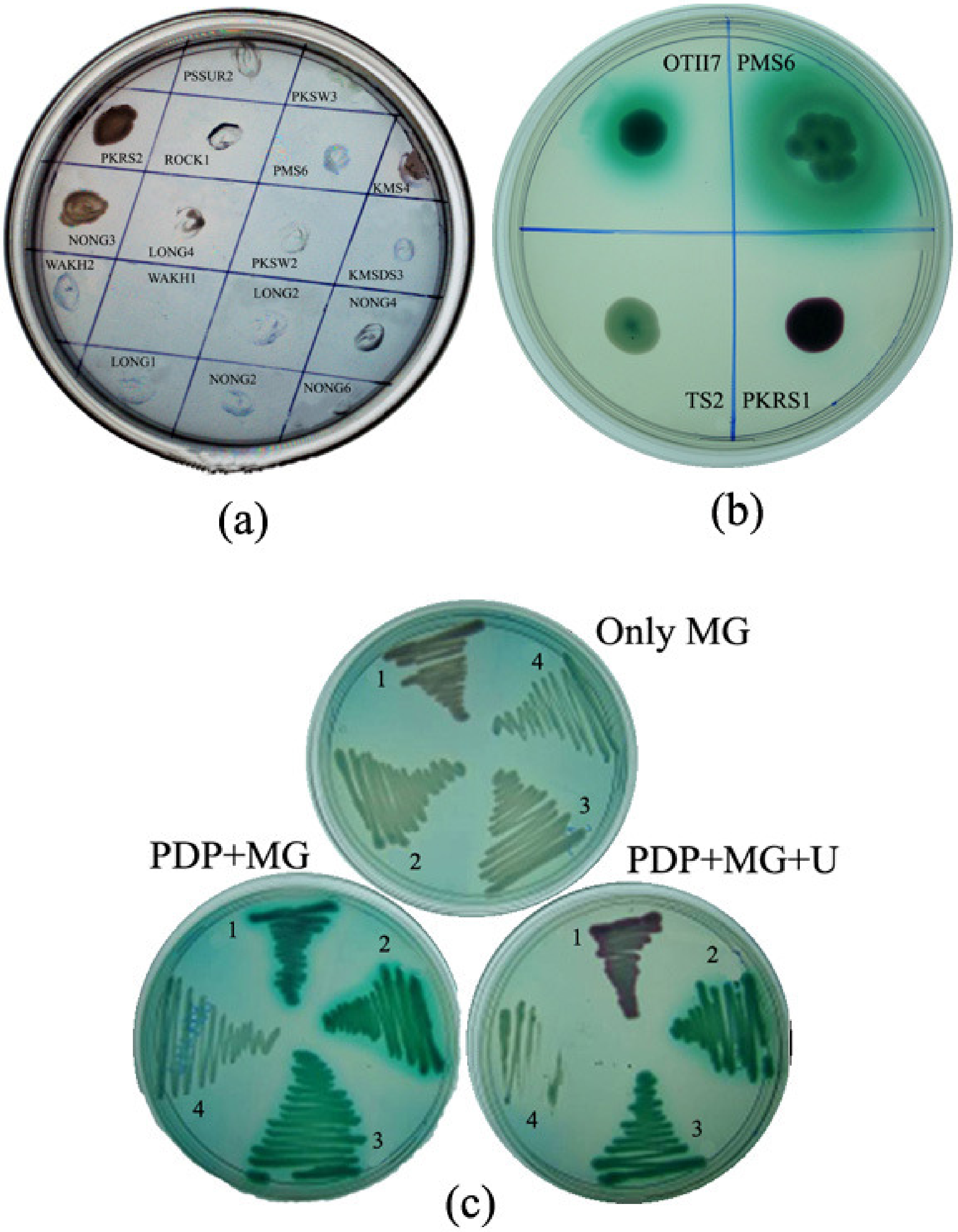

Fig. S3 Analysis of phosphatase activity of identified isolates on histochemical plates. (a) Number of colonies patched on the histochemical plate for random and fast selection of positive isolates. (b) Patching of individual isolate on histochemical plate for detailed study as in this figure it explains as, OTII7 shows intra and extracellular phosphatase(s), PMS6 has extracellular phosphatase(s), PKRS1 portrays intracellular phosphatase and TS2 may not have an active phosphatase or if at all it has, it may have a stationary phase inducible phosphatase (c) Comparative test for positive isolates in various conditions, The plates containing only MG and no phosphate source (PDP) does not show any bluish-green colouration around the colony while when phosphate source is added the three bacteria except TS2 showed bluish green concentration and as these bacteria can tolerate U, they were tested for their phosphatase activity in its presence which has shown similar reading as in PDP-MG plates apart from diminished growth of cultures. The positive screening on PDP-MG plates without U confirms that the phosphatase enzyme is expressed constitutively and is not produced only when there is a stress factor.

Table S1 Presence of P_{IB} type ATPase gene in the multi-metal resistant Domiasiat isolates and their closest identified match in the NCBI GenBank database

Isolates (Accession No.)[a]	Closest identified match in NCBI GenBank with similarity percentage[b]
***cadA* gene**	
Bacillus halmapalus LONG2 (JN222559)	Cadmium-transporting ATPase, *Geobacillus kaustophilus* HTA426, (YP 146205), 64%
Bacillus thuringiensis KMSZP5 (JN034438)	Cadmium-transporting ATPase, *Lysinibacillus sphaericus* C3-41, (YP 001699920), 94%
Bacillus isronensis PKSW3 (JN034437)	Cation transport ATPase, *Solibacillus silvestris* StLB046, (BAK 15053.1), 94 %<br>Cadmium-transporting ATPase, *Lysinibacillus sphaericus* C3-41, (YP 001696058.1), 67 %
Lysinibacillus xylanilyticus WAKH2 (JN222556)	Cadmium-transporting ATPase CadA, *Lysinibacillus fusiformis* ZC1, (ZP 07051554), 81 %
Lysinibacillus xylanilyticus NONG2 (JN222557)	Cadmium-transporting ATPase CadA, *Lysinibacillus fusiformis* ZC1, (ZP 07051554), 85 %
Lysinibacillus xylanilyticus LONG1 (JN222558)	Cadmium-transporting ATPase CadA, *Lysinibacillus fusiformis* ZC1, (ZP 07051554), 82 %
Lysinibacillus xylanilyticus KMSDS4 (JN034436)	Cadmium-transporting ATPase, *Lysinibacillus sphaericus* C3-41, (YP 001699920), 95%
***copA* gene**	
Pseudomonas koreensis OT6 (JN034439)	Copper-transporting P-type ATPase, *Providencia rettgeri* DSM 1131, (ZP 06125459), 92%
Arthrobacter mysorens RSBA2 (JN634869)	Cation-transporting ATPase, *Arthrobacter arilaitensis* Re117, (YP 003918270), 78%<br>Copper-translocating P-type ATPase, *Arthrobacter aurescens* TC1, (YP 949762), 55%
***zntA* gene**	
Paenibacillus pabuli PSS2 (JN034435)	Zinc-exporting ATPase, *Sphingobacterium spiritivorum* ATCC 33861, (ZP 07080472), 66%
Chryseobacterium culicis PMSZPI (JN034431)	Zinc-exporting ATPase, *Chryseobacterium gleum* ATCC 35910, (ZP 07084814), 92%
Sphingobacterium siyangense KMSDrP1 (JN034432)	Zinc-exporting ATPase, *Sphingobacterium spiritivorum* ATCC 33861, (ZP 07080472), 66%
Sphingobacterium siyangense KMSDrP2 (JN034433)	Zinc-exporting ATPase, *Sphingobacterium spiritivorum* ATCC 33861, (ZP 07080472), 66%
Sphingobacterium siyangense KMSDrP3 (JN034434)	Zinc-exporting ATPase, *Sphingobacterium spiritivorum* ATCC 33861, (ZP 07080472), 66%
Sphingobacterium siyangense KMSZPIII (JN634870)	Zinc-exporting ATPase, *Chryseobacterium gleum* ATCC 35910, (ZP 07089093), 76%
Bacillus cereus KMSII3 (JN222560)	Zinc-transporting ATPase, *Bacillus thuringiensis* serovar *israelensis* ATCC 35646, (ZP 00741169), 99%

[a] Accession number obtained from NCBI GenBank for identified P_{IB}-type ATPase gene are given in parenthesis

[b] Closest identified match (phylogenetic neighbours) of the identified gene in NCBI GenBank database. Numbers in parenthesis indicate their GenBank accession numbers followed by maximum identity percentage
